# An Unusual Case Presentation of Emphysematous Cystitis

**DOI:** 10.7759/cureus.10532

**Published:** 2020-09-18

**Authors:** Ariana Ganji, Ali A Malhi, Sarkis Kouyoumjian

**Affiliations:** 1 Emergency Medicine, Wayne State University Detroit Medical Center, Detroit, USA

**Keywords:** emphysematous cystitis, computer tomography, plain radiography

## Abstract

Emphysematous cystitis (EC) is a rare infection characterized by gas formation in the bladder wall. We present an unusual case of emphysematous cystitis of a 37-year-old female patient with pneumaturia, vomiting, nausea and abdominal pain. EC was diagnosed on a computed tomography (CT) scan which demonstrated abnormal bladder wall thickening that contained air. Urinalysis was positive for hematuria, bacteria and an elevated leukocyte esterase which further supported the diagnosis. This patient's atypical symptoms and lack of other risk factors that are commonly seen in patients with EC highlight the need for vigilance when assessing for EC.

## Introduction

Emphysematous cystitis (EC) is a rare condition that is characterized by gas in the bladder wall and/or lumen. It is often caused by gas-producing pathogens such as *Escherichia coli* [1]. Although research demonstrates that abdominal tenderness and pain are common symptoms among patients, there is currently a paucity of specific clinical symptoms that can be used to diagnose EC [2]. Thus, physicians rely on risk factors to assist in screening patients for EC, which include: older age (over 60 years old), diabetes mellitus, history of recurrent urinary tract infections, or neurogenic bladder [3]. However, many patients present atypically. Herein, we present a case of a 37-year-old female patient with pneumaturia, vomiting, nausea, and abdominal pain who was diagnosed with emphysematous cystitis. 

## Case presentation

A 37-year-old African-American female presented to the emergency department (ED) complaining of abdominal pain, nausea, vomiting, diaphoresis, chills, and burning with urination. The patient stated she had dysuria and “bubbles in her urine”. She also stated that the pain was 10 (on a scale of 1-10) and described the quality as “crampy”. The patient had a past medical history of diabetes mellitus, gastritis, hypertension, and kidney stone. She also had a past surgical history of a Caesarian section. Additionally, the patient was a regular cigarette smoker, marijuana user, and alcohol drinker. 

Physical exam revealed a blood pressure of 173/80 mmHg, a body temperature of 36.6 degrees Celsius, a respiratory rate of 20/min, and a pulse rate of 100/min. The patient was six feet tall and weighed 125 pounds; this was a significant decrease from her weight of 187 pounds less than eight months ago.

Urine analysis was performed, which revealed hematuria, bacteria, and an elevated leukocyte esterase; this suggested a possible urinary tract infection. Complete cell count revealed a white blood cell (WBC) count of 8.0 cells/mcL, hemoglobin of 15.6 gm/DL, red blood cell (RBC) count of 4.28 cells/mcL, and platelet count of 214,000 cells/mcL. The basic metabolic profile revealed a low potassium level of 3.3mMol/L, and an elevated glucose level at 392 mg/dL with an anion gap of 15.

**Figure 1:**
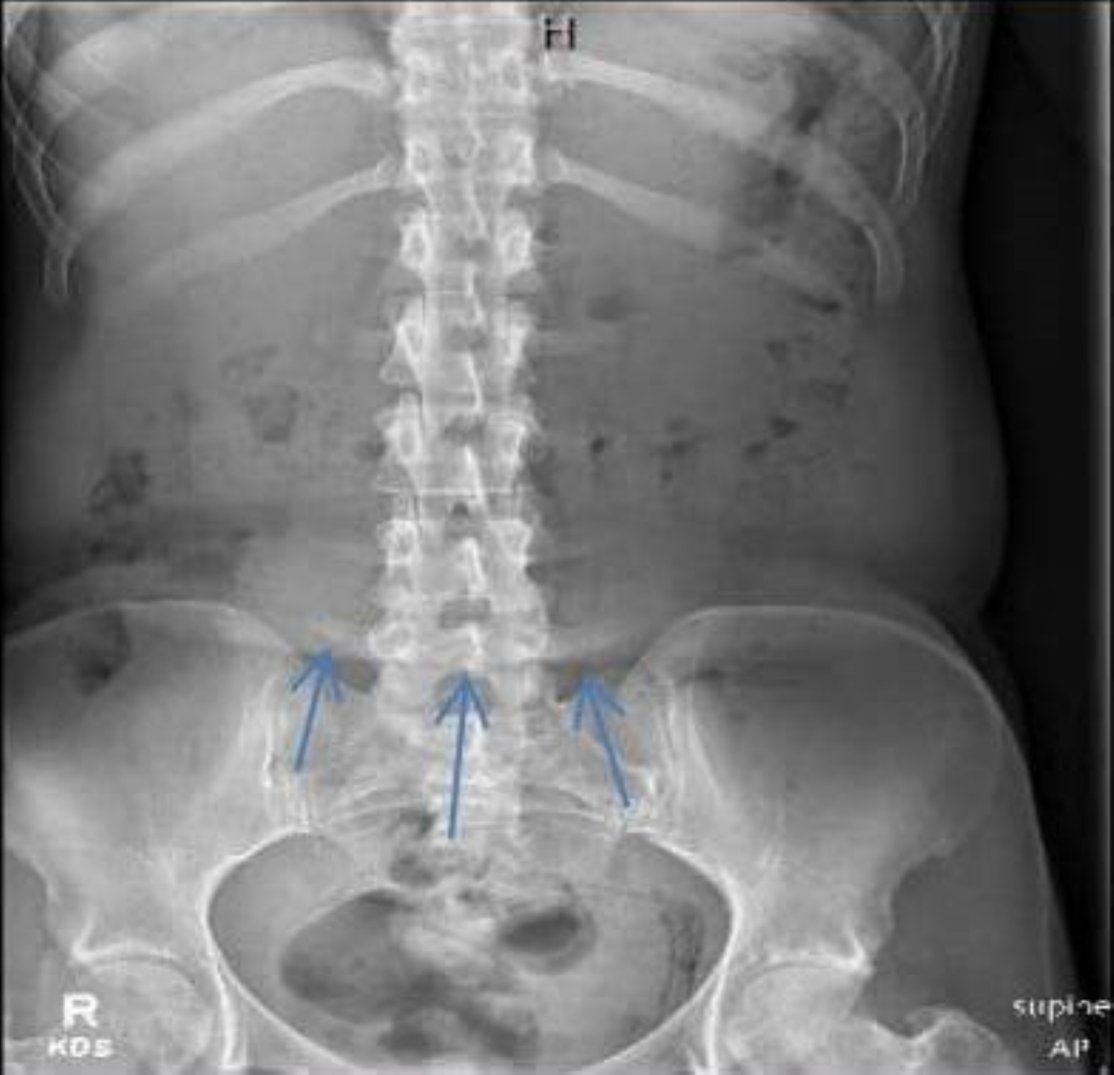
Abdominal X-ray demonstrating curvilinear lucencies in the pelvis

**Figure 2:**
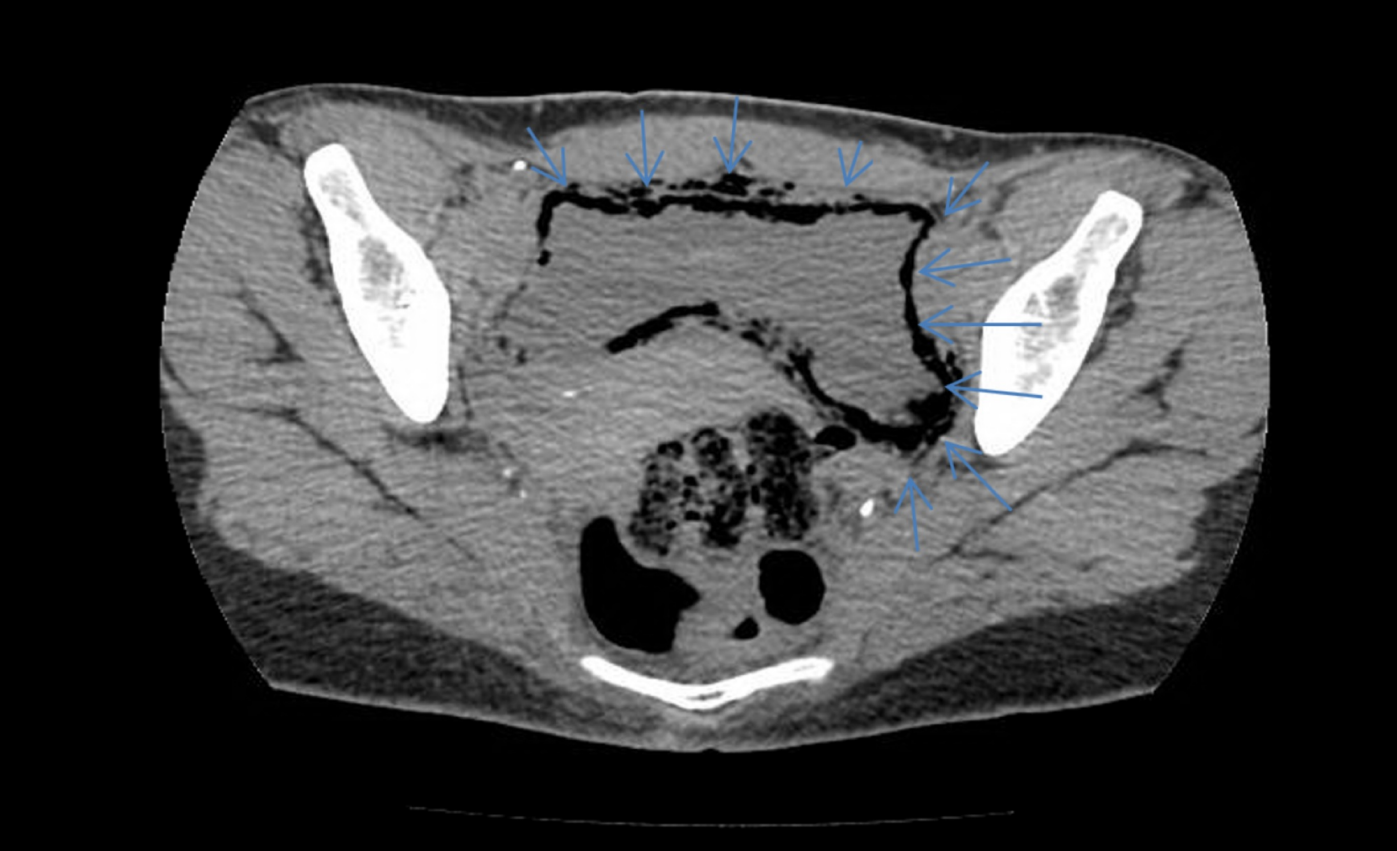
A CT scan of the pelvis and abdomen without contrast indicating abnormal bladder wall thickening that contained air

An abdominal X-ray was performed to evaluate ileus or obstruction, and it revealed curvilinear lucencies in the pelvis (Figure 1). Computed tomography (CT) scan was recommended to evaluate for EC. A CT scan of the pelvis and abdomen without contrast indicated that the patient had abnormal bladder wall thickening that contained air (Figure 2). The diagnosis of acute emphysematous cystitis was made after these multiple indications.

The patient was started on intravenous fluid hydration and admitted to the hospital. The patient was started on vancomycin, cefepime, metronidazole, and a foley catheter was recommended but refused by the patient. A urine culture was ordered to determine the pathogen and the appropriate antibiotic regimen. However, the patient left against medical advice prior to the results.

## Discussion

Very sparse information regarding emphysematous cystitis has been reported. Thomas et al. (2007) reviewed 135 cases of emphysematous cystitis from 1956 to 2006 using an English literature review search [1]. Over 2/3 of reported cases were diabetic, the mean age was 61.9 years, and 62% were women. However, the clinical presentation of emphysematous cystitis varied among 135 cases. Many patients possessed symptoms that ranged from pneumaturia to severe sepsis with an acute abdomen. Furthermore, seven percent of the patients were completely asymptomatic. Among the cases, different types of bacteria and fungi were identified within the urine of the patient. *Escherichia coli* *(E. coli)* was seen in 58% of cases, making it the most commonly isolated bacterial or fungal organism found within the urine of the patients. Other organisms found include* Staphylococcus aureus, Proteus mirabilis, Candida albicans, Klebsiella pneumoniae, Enterococcus faecali, *and others.

Grubber et al. (2007) conducted an English-language literature review of EC cases from 1986 to 2006 [2]. They examined 53 cases and reported similar demographic findings as Thomas et al. The average age was 67 years old +/- 15 years. Diabetes mellitus was present in 72% of cases and 56% of patients were female. The authors also reported variability in symptoms but determined that abdominal pain was the most common symptom, occurring in over 80% of patients. Other symptoms included gross or microhematuria, which was present in 82.3% of patients, and tenderness of the abdomen that was present in 65.6% of cases. However, only 53% of cases reported classic urinary tract infection (UTI) symptoms such as urinary frequency, urinary urgency, and dysuria. Our patient reported less common symptoms of vomiting, chills, and nausea, which was present in 47%, 25%, 60% of patients, respectively. Pneumaturia (present in our patient who described her symptom as "bubbles in the urine") that was recorded in the history was present in 26.7% of cases. Additionally, 65.6% of cases reported pneumaturia after catheterization. Amano and Shimizu (2014) completed a more recent review of the literature, examining both English and Japanese cases from 2007- 2013 [3]. They reached similar conclusions regarding the demographics of EC compared to past reviews. They reported a mean age of 68 years and 72 years in Japan and outside of Japan, respectively. The majority of patients were female and diabetic. A review of the literature completed by Schicho et al. (2017) also reached similar conclusions with elderly female diabetics representing the majority of patients [4]. Neither study examined clinical symptoms.

Due to the variability of symptoms, it is difficult to accurately diagnose EC by clinical presentation alone. The most important tools for diagnosing are CT and/or plain conventional abdominal radiography. CTs are preferred because plain films are less sensitive than CT imaging in demonstrating the amount of gas formation. Additionally, CT scans can exhibit the severity of the condition and rule out other sources of pelvic air [3]. Physicians may also utilize bladder ultrasounds; however, they have lower sensitivity than CT scans. It is noted that it may be useful for serial monitoring in patients who have demonstrated clinical amelioration [5]. However, the air within the bladder wall or lumen could be attributed to a fistula (most commonly colovesical), trauma, colon carcinoma, or instrumentation - these are all other examples of differential diagnoses that should be considered [6]. Additionally, it may be helpful to perform a urinalysis and gram staining of urine cultures to discover the causative pathogen and determine a suitable antibiotic regimen [3]. It is speculated that *E. coli* ferments the glucose in the urine, which may explain the link between diabetics and EC [7].

## Conclusions

The patient discussed in this report was significantly younger than the typical age of onset for EC and displayed unconventional symptoms such as vomiting and chills. Because no clear clinical picture can be used alone to accurately diagnose EC, physicians often rely on historical risk factors (over 60 years of age, diabetes mellitus, history of recurrent urinary tract infections, or neurogenic bladder) to assist in the making of the diagnosis. Some physicians may have not considered this patient for a diagnosis of EC, as the typical age of onset is almost 30 years greater than this patient's age. This case highlights the importance of physicians remaining vigilant and screening for emphysematous cystitis even in atypical patients. Pneumaturia appears to be a highly specific symptom, emphasizing the consideration of EC in the differential diagnosis for a patient presenting with "bubbles in the urine". Computed tomography and/or plain conventional abdominal radiography are tools that can greatly aid clinicians in their assessment of these patients.
